# Association of *IL10* and *TGFB* single nucleotide polymorphisms with intervertebral disc degeneration in Iranian population: a case control study

**DOI:** 10.1186/s12881-018-0572-2

**Published:** 2018-04-10

**Authors:** Sara Hanaei, Sina Abdollahzade, Maryam Sadr, Mohammad Hossein Mirbolouk, Alireza Khoshnevisan, Nima Rezaei

**Affiliations:** 1 Molecular Immunology Research Center, Tehran University of Medical Sciences, Tehran, Iran; 2 Department of Neurosurgery, Shariati Hospital, Tehran University of Medical Sciences, Tehran, Iran; 3 Research Center for Immunodeficiencies, Children’s Medical Center, Tehran University of Medical Sciences, Dr Qarib St, Keshavarz Blvd, Tehran, 14194 Iran; 4 Department of Immunology, School of Medicine, Tehran University of Medical Sciences, Tehran, Iran; 5 Network of Immunity in Infection, Malignancy and Autoimmunity (NIIMA), Universal Scientific Education and Research Network (USERN), Tehran, Iran

**Keywords:** Intervertebral disc degeneration, Single nucleotide polymorphism, Interleukin 10, Transforming growth factor β

## Abstract

**Background:**

Considered as one of the major causes of low back pain, Intervertebral disc degeneration (IVDD) is caused by several genetic and environmental factors. As inflammation plays an important role in disc degeneration, the genetic changes in both inflammatory and anti-inflammatory genes may play causative roles in IVDD as well. Therefore, the interactions between inflammatory and anti-inflammatory cytokines and also other components of disc matrix would determine the degree of tissue destruction in disc degeneration. However, there is still controversy regarding the exact role of inflammation and disc homeostasis imbalance in pathophysiology of IVDD. Therefore, current study was conducted to investigate the role of IL-10 and TGF-β single nucleotide polymorphisms (SNP) in Iranian IVDD patients.

**Methods:**

Seventy-six IVDD patients and 140 healthy controls were enrolled in this study. Genomic DNA from peripheral leukocytes was tested for 3 SNPs in IL10 (L-10 -1082G/A (rs1800896), IL-10 -819C/T (rs1800871), IL-10 -592A/C (rs1800872)) and 2 SNPs in TGF-β (TGF-β Codon 10 C/T (rs1982037), and TGF-β Codon 25 C/T (rs1800471) genes through PCR-SSP method. The extracted genomic DNA was genotyped for the aforementioned SNPs of interest using specific primers, which were coated in the cytokines KITs and based on the PCR-SSP method for sequencing.

**Results:**

The ‘T’ allele of IL-10 -819C/T and the ‘C’ allele of IL-10 -592A/C were more prevalent among patients, whereas the ‘C’ and ‘A’ alleles of respective SNPs were significantly more frequent in controls. The genotypes including ‘CT’ of IL-10 -819C/T, ‘CA’ of IL-10 -592A/C, and ‘GA’ of IL-10 -1082A/G were more common among patients, while the ‘CC’ genotype of both IL-10 -819C/T and IL-10 -592A/C SNPs were more frequent in controls. In addition, the IL-10 haplotypes including ‘ACC’, ‘ATA’, and ‘ACA’ were significantly associated with disease. Meanwhile, the ‘TC’ haplotype of TGF-β was more common among patients as well.

**Conclusions:**

The IL-10 SNPs were significantly associated with IVDD in Iranian population; which proposes that genomic alterations of anti-inflammatory cytokines could lead to homeostasis imbalance in intervertebral discs and degenerative changes.

## Background

Comprising a disabling age-related health problem among middle-aged population, intervertebral disc degeneration (IVDD) is of the main causes of low back pain. The first pathologic changes in discs would start in adolescence age. Although degenerative disc disease could be asymptomatic in early stages, the patients would experience an spectrum of low back pain with disease progression [1]. The homeostasis imbalance in both inner and outer layers of intervertebral disc [2] could lead to morphological changes in disc space and endplates [3]. Considering the multifactorial nature of disease, both genetic and environmental factors, as well as their interactions, may play causative roles in etiology of IVDD. In addition to alteration in the expression pattern of numerous genes, gene mutations and single nucleotide polymorphisms (SNP) could participate in etiology of degenerative changes in this disease [1, 4].

Local concentration of inflammatory mediators, produced by inflammatory immune cells, could lead to local inflammation in degeneration process. The interactions between pro-inflammatory cytokines (IL-1, IL-6, and TNF-α,) and anti-inflammatory cytokines (as interleukin 10 (IL-10),) together with other etiologic risk factors (other genes, mechanical loads, etc.) would determine the extreme of destruction in disc through degeneration process [5]. As an anti-inflammatory mediator, IL-10 has shown an inhibitory effect on production of pro-inflammatory cytokines. Produced by a number of immune cells, the expression level of IL-10 has been enhanced during disc degeneration [5, 6], specifically in bone and connective tissue structures near the disc [5]. Degenerated discs of both animal model of disc degeneration [7] and human samples [6] have similarly supported the up-regulation of this anti-inflammatory cytokine. Due to the regulatory role of IL-10 gene promoter, nucleotide substitution(s) in this region, would lead to alterations in transcription and consequently expression of this gene [5], therefore, IL-10 promoter polymorphisms could participate in etiology of IVDD as a genetic risk factor.

Mainly participating in matrix production and metabolic regulation [8], transforming growth factor β (TGF-β) belongs to a large family of anabolic cytokines which are found in intervertebral discs as well [9, 10]. Comparing degenerated discs with herniated ones has indicated higher expression level of TGF-β in degenerated discs [11]. On the other hand, the expression level of TGF-β has remarkably decreased in higher degrees of disc degeneration [12], which is in contrary with other findings indicating higher expression levels in higher degeneration grades [11, 13].

Although several genetic investigations have been reported in IVDD, the exact roles of inflammatory mediators and their regulators in pathophysiology of IVDD are not clearly defined. Besides, we studied association of IL10 and TGF-β with different diseases [14–20], but these two genes have not been investigated in IVDD Iranian population. So, as the increasing burden of IVDD in socioeconomic condition of individuals and therefore societies, and accordingly to different gene pools in different ethnicities, current study was conducted to determine the association between IL-10 and TGF-β SNPs with IVDD among Iranian population.

## Methods

### Study design and setting

In order to investigate the association between IVDD and SNPs in IL10 and TGF-β genes, the current case-control study was designed according to the STROBE guideline for observational studies. Meanwhile, the study was conducted in Molecular Immunology Research Center, Tehran University of Medical Sciences (TUMS), Tehran, Iran.

### Patient selection

Over a two-year study period, adult patients with at least 6-month history of chronic low back pain who were diagnosed with intervertebral disc degeneration by two independent neurosurgeons, irresponsive to medical treatment and indicated for surgical intervention were included in this study. All the patients had experienced mechanical low back pain and buttock pain in addition to radicular pain to lower limb(s), which indicated compression of nerve root(s) by degenerated herniated disc. Moreover, the MRI images indicated protruded and/or extruded intervertebral discs with degenerative changes. The patients with other etiologies of low back pain including trauma, spine neoplasm, spondylolysis, spondylolisthesis, ankylosing spondylitis, spondyloarthropathies, and other systemic inflammatory diseases were not eligible for this genetic study and therefore were excluded. None of the eligible patients fulfilled diagnostic criteria for any of the aforementioned inflammatory diseases. The 76 patients were compared with 140 adult healthy subjects (70 males and 70 females) as previously described [21]. The eligibility criteria for control group included healthy individuals with no current or previous history of chronic low back pain and also other systemic inflammatory diseases. The physical examination of all control subjects were in normal ranges in order to rule out the symptomatic IVDD and nerve root compression. Those subjects who were asymptomatic but have previously experienced periods of chronic low back pain or had history of lumbar surgery were excluded from this study. The participants were requested to sign informed consent forms prior to blood testing; and the study was approved by the Ethics Committee of Tehran University of Medical Sciences (TUMS).

### Clinical data

Demographic characteristics and clinical data including level of lesion, pre-operative, 2-month and 6-month post-operative visual analogue scale (VAS) and Oswestry disability index (ODI) were either obtained from patients’ files or clinically evaluated; and then recorded in data collection forms.

### Blood sampling and DNA extraction

A total volume of 5cm^3^ peripheral blood was obtained from all patients and controls in EDTA-containing falcon tubes in order to prevent coagulation. The samples were stored at -20°C prior to DNA extraction. The DNA was extracted from peripheral leukocytes through Phenol-Chloroform method, as previously described [22]. The extracted DNA was then stored at -20°C prior to polymerase chain reaction (PCR). The optical density and the 260/280 ratio were measured in order to assess DNA concentration and quality.

### Sequence specific prime PCR (PCR-SSP)

A total volume of 10 μl solution, comprised of 1.43 μl Master Mix, 3.43 μl dH_2_O, 0.57 μl DNA, and 0.04 μlTaq enzyme, was placed in each well of 96-well reaction plate of Cytokine CTS-PCR-SSP TRAY KIT (University Clinic Heidelberg, Heidelberg, Germany) and the reactions were consecutively performed accordingly to the respective protocol [16]. The plates were placed into the PCR machine and the reactions were performed as 1 cycle of 94° for 3 min, followed by 30 cycles of 94° for 30 s, 54° for 30 s, and 72° for 30 s. Finally, there was one cycle of 72° for 5 min. In brief, the extracted genomic DNA was genotyped for 5 SNPs in IL10 and TGF-β using specific primers which were coated in the cytokines KITs and based on the PCR-SSP method for sequencing. SNPs of IL-10 and TGF-β including IL-10 -1082G/A (rs1800896), IL-10 -819C/T (rs1800871), IL-10 -592A/C (rs1800872), TGF-β Codon 10 C/T (rs1982037), and TGF-β Codon 25 C/T (rs1800471) have been tested and compared in cases and controls.

### Statistical analysis

Using the PASS 11 software, a sample size of 50 would achieve 81% power to detect an effect size (W) of 0.40 using a 1 degree of freedom Chi-Square Test with a significance level (alpha) of 0.05. Quantitative variables as age, pre and post-operative VAS and ODI are reported as mean ± SD. The qualitative variables as sex, lesion level, allele, genotype, and haplotype distributions are reported as frequencies and the comparison between allele, genotype, or haplotype frequencies in cases and controls have been determined through Chi^2^ test. Association between genotype frequencies and VAS or ODI mean differences (MD) are measured through One-way ANOVA test and association between allele distributions and VAS or ODI mean differences are measured through independent sample T-test. A *P* value of less than 0.05 was considered as significant in all tests. The Online Encyclopedia for Genetic Epidemiology studies was used in order to calculate Hardy-Weinberg Equilibrium (HWE) [23].

## Results

### Patients’ characteristics

Demographic and clinical characteristics of patients are described in detail in Table 1. The patients included 61.8% males and 38.2% females with mean age of 39.08 ± 10.62 years ranging from 19 to 62 years and the most common level of degeneration was L4-L5 (43.7%). Visual analogue scale and Oswestry Disability Index (ODI) have been assessed in 66 patients. The average pre-operative VAS score was 8.37 ± 0.73, followed by 2-month and 6-month post-operative scores as 3.83 ± 1.15 and 2.59 ± 1.00 respectively. Mean pre-operative ODI was 37.80 ± 3.96, followed by 2-month and 6-month post-operative ODI as 17.87 ± 4.91 and 12.01 ± 3.63 respectively. The controls included 70 females and 70 males who were not remarkably different from patients (X^2^ = 2.78, *P* = 0.09)

**Table 1:** Patients’ characteristics

Variable	IVDD Patients (*N* = 76)	Healthy Controls (*N* = 140)	*P*-Value
Age			
Mean ± SD	39.08 ± 10.62 years		
Range	19–62 years		
Sex			
Male N (%)	47/76 (61.8%)	70/140 (50%)	0.1159
Female N (%)	29/76 (38.2%)	70/140 (50%)	
Level of degeneration			
L2/L3	3/71 (4.2%)	–	–
L3/L4	5/71 (7%)		
L4/L5	31/71 (43.7%)		
L3/L4 & L4/L5	3/71 (4.2%)		
L5/S1	25/71 (35.2%)		
L4/L5 & L5/S1	4/71 (5.6%)		
VAS (Mean ± SD) (*N* = 66)			
Pre-operative	8.37 ± 0.73	–	–
2-month post-operative	3.83 ± 1.15		
6-months post-operative	2.59 ± 1.00		
ODI (Mean ± SD) (N = 66)			
Pre-operative	37.80 ± 3.96	–	–
2-month post-operative	17.87 ± 4.91		
6-months post-operative	12.01 ± 3.63		

### The association of IL-10 and TGF-β single nucleotide polymorphism with intervertebral disc degeneration

**Fig. 1:**
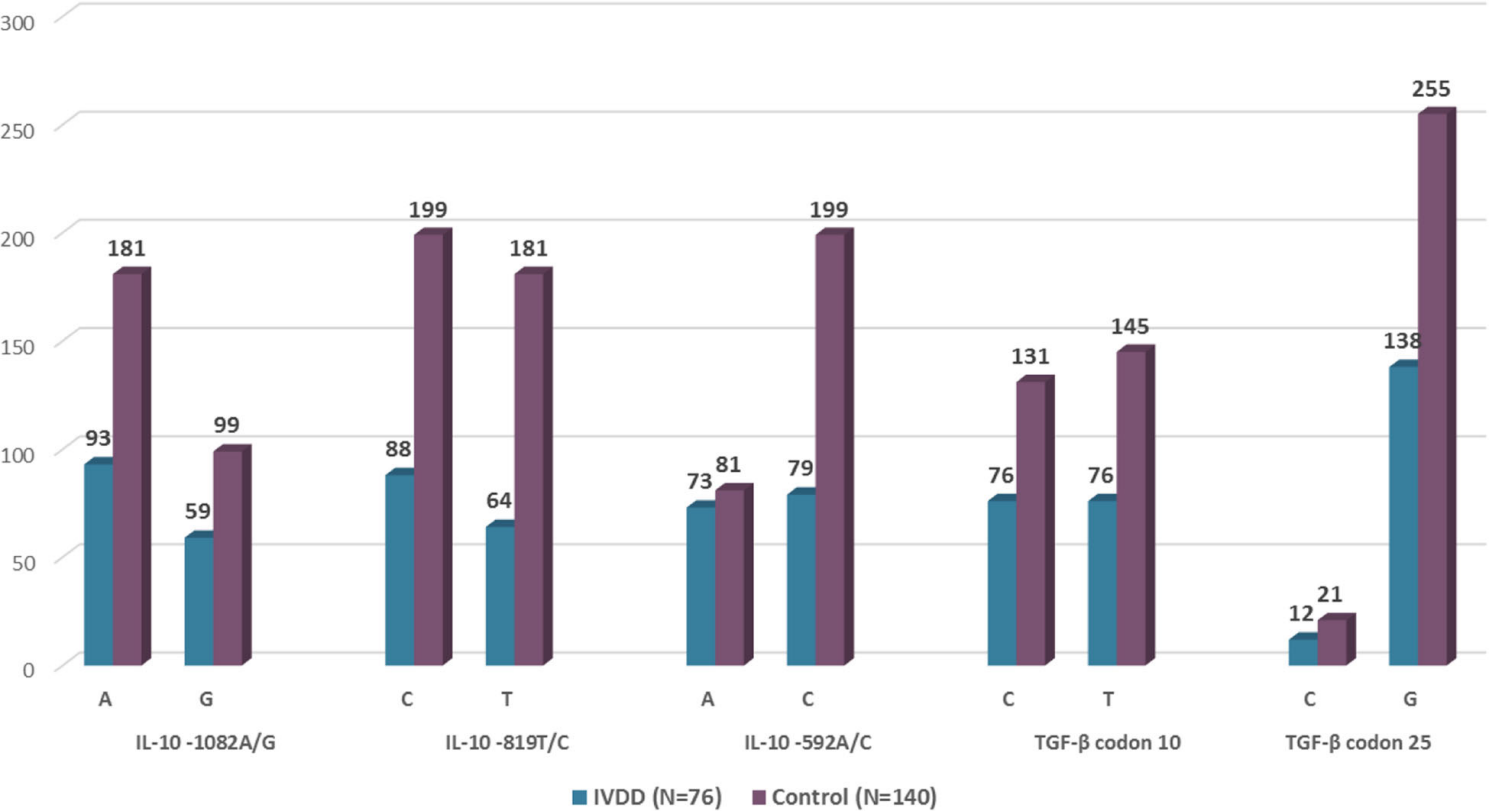
Allele Frequencies of IL-10 and TGF-β SNPs in IVDD and Controls

**Fig. 2:**
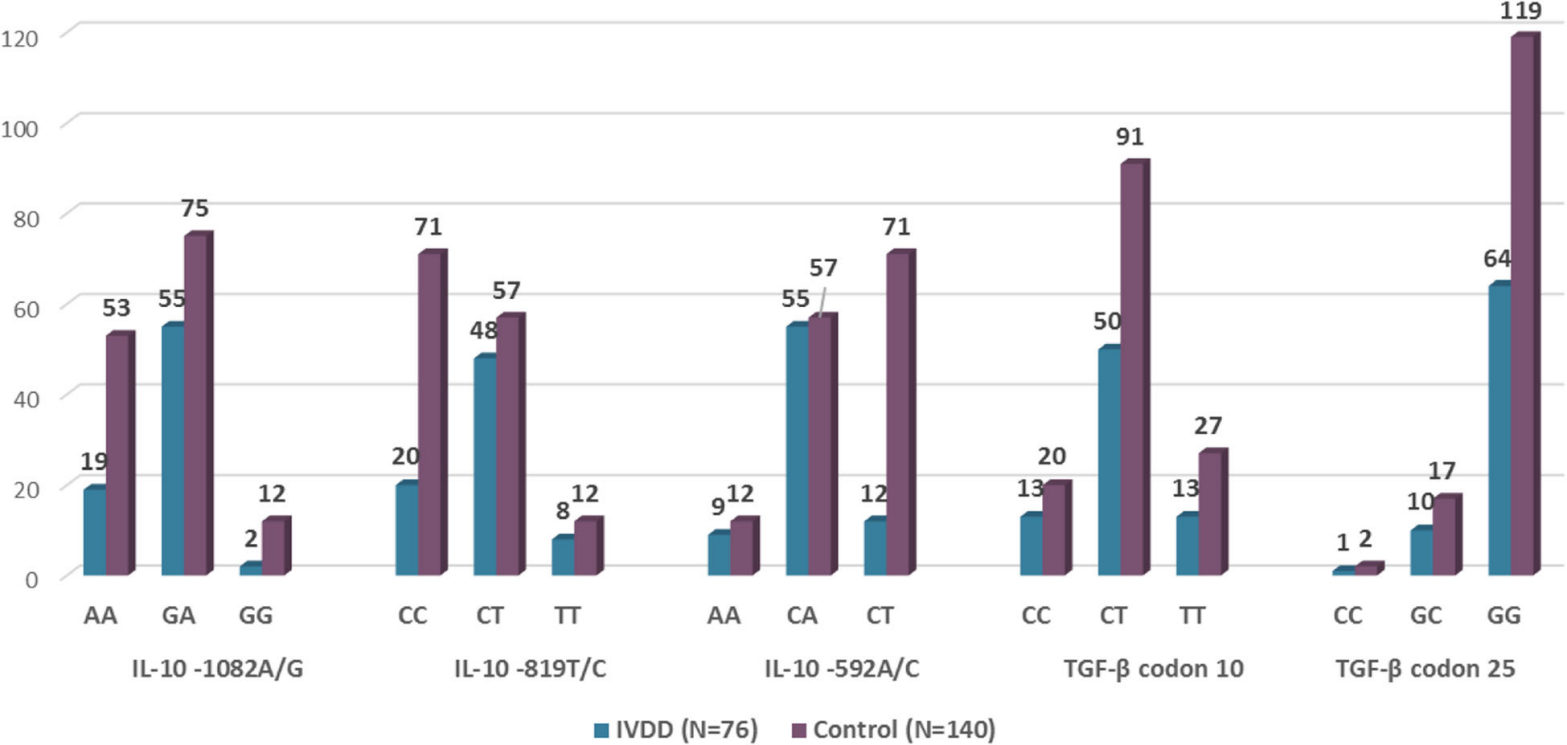
Genotype Frequencies of IL-10 and TGF-β SNPs in IVDD and Controls

Of the investigated polymorphisms, the IL-10 SNPs have indicated significantly different distributions in cases and controls. While the ‘T’ allele of IL-10 -819C/T has been 1.78 times more common among IVDD subjects (OR = 1.78, 95% CI = 1.17–2.70, *P* = 0.008), the ‘C’ allele of this SNP has indicated a protective role for IVDD (OR = 0.67, 95% CI = 0.37–0.85, P = 0.008), and the ‘CC’ genotype has been more common in healthy individuals as well (OR = 0.35, 95% CI = 0.19–0.64, *P* = 0.001). The ‘CT’ genotype, however, has been 2.49 times more frequent in IVDD patients and therefore increased the disease susceptibility. This may indicate that whereas presence of ‘T’ allele increases the IVDD risk; homozygosity for ‘C’ could decrease susceptibility to disease (Table 2, Figs. 1 and 2).

**Table 2:** IVDD-Control allele and genotype frequency comparison

Cytokine	Position	Alleles/ Genotypes	IVDD	Control	Hardy-Weinberg equilibrium X^2^ *P* value	*P*-Value	OR	95% CI
			(*N* = 76)	(*N* = 140)				
			*N* (%)	*N* (%)				
TGF-β	Codon 10	C	76 (50)	131 (47.5)	–	0.69	1.11	0.74–1.65
		T	76 (50)	145 (52.5)	–	0.69	0.90	0.60–1.35
		CC	13 (17.1)	20 (14.5)	5.81 (0.015)	0.69	1.22	0.57–2.61
		CT	50 (65.8)	91 (65.9)		1.00	0.99	0.55–1.79
		TT	13 (17.1)	27 (19.6)		0.72	0.85	0.41–1.76
TGF-β	Codon 25	C	12 (8)	21 (7.6)	–	1.00	1.06	0.50–2.21
		G	138 (92)	255 (92.4)	–	1.00	0.94	0.45–2.00
		CC	1 (1.3)	2 (1.5)	2.12 (0.14)	1.00	0.92	0.08–10.30
		GC	10 (13.3)	17 (12.3)		1.00	1.09	0.47–2.53
		GG	64 (85.3)	119 (86.2)		1.00	0.93	0.42–2.07
IL-10	-1082	A	93 (61.2)	181 (64.6)	–	0.53	0.86	0.57–1.29
		G	59 (38.8)	99 (35.4)	–	0.53	1.16	0.77–1.75
		AA	19 (25)	53 (37.8)	4.14 0.041)	0.07	0.55	0.29–1.02
		GA	55 (72.4)	75 (53.6)		0.009	2.27	1.24–4.15
		GG	2 (2.6)	12 (8.6)		0.15	0.29	0.06–1.32
IL-10	-819	C	88 (57.9)	199 (71.1)	–	0.008	0.56	0.37–0.85
		T	64 (42.1)	81 (28.9)	–	0.008	1.78	1.17–2.70
		CC	20 (26.3)	71 (50.7)	0.01 (0.92)	0.001	0.35	0.19–0.64
		CT	48 (63.2)	57 (40.7)		0.002	2.49	1.40–4.44
		TT	8 (10.5)	12 (8.6)		0.81	1.25	0.49–3.22
IL-10	-592	A	73 (48)	81 (28.9)	–	0.000	2.27	1.51–3.42
		C	79 (52)	199 (71.1)	–	0.000	0.44	0.29–0.66
		AA	9 (11.8)	12 (8.6)	15.36 (<0.0001)	0.47	1.43	0.57–3.57
		CA	55 (72.4)	57 (40.7)		0.000	3.81	2.08–6.98
		CC	12 (15.8)	71 (50.7)		0.000	0.18	0.09–0.37

The ‘A’ allele of IL-10 -592 A/C has been 2.27 times more prevalent in patients (OR = 2.27, 95% CI = 1.51–3.42, *P* < 0.001), whereas the ‘C’ allele has protected the subjects against disease (OR = 0.44, 95% CI = 0.29–0.66, *P* < 0.001). Moreover, while the ‘CA” genotype has increased the IVDD risk for 3.81 times (OR=3.81, 95% CI=2.08-6.98, *P*<0.001), the ‘CC’ genotype have been protective against IVDD (OR = 0.18, 95% CI = 0.09–0.37, *P* < 0.001). This may indicate that while presence of ‘A’ allele could be considered as a risk factor for IVDD, homozygosity for ‘C’ could be protective (Table 2, Figs. 1, and 2).

The ‘GA’ genotype of IL-10 -1082G/A has been 2.27 times more frequent among patients (*P* = 0.009), however other allele or genotype distributions of IL-10 were not remarkably different from healthy subjects.

On the other hand, however, none of investigated TGF-β SNPs (codons 10 and 25) has shown remarkable association with IVDD, through either allele or genotype distribution (Table 2, Figs. 1 and 2)

### Association between haplotypes of IL-10 and TGF-β single nucleotide polymorphisms and intervertebral disc degeneration

**Fig. 3:**
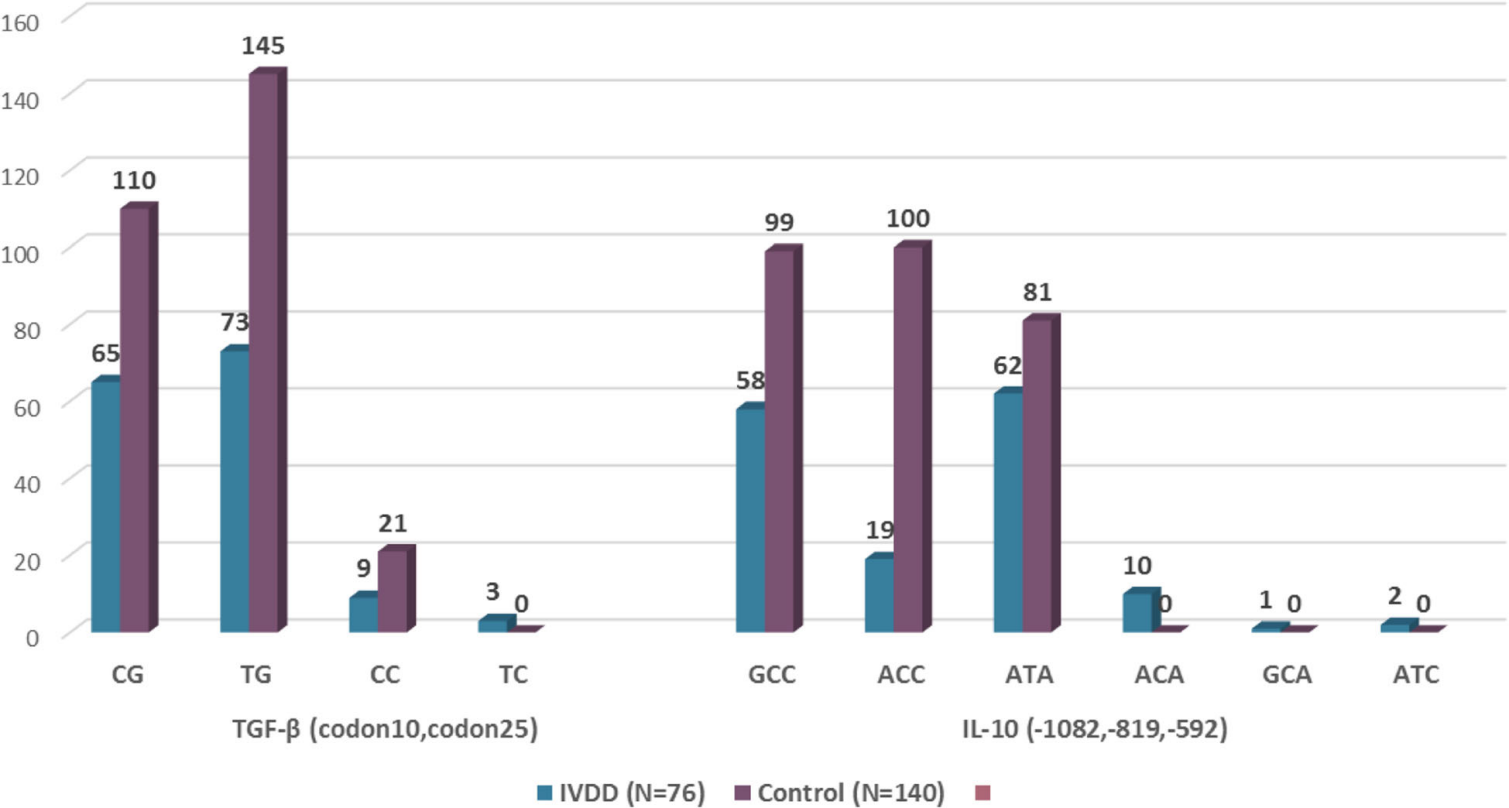
Haplotype Frequencies of IL-10 and TGF-β SNPs in IVDD and Control

Considering co-inheritance of SNPs, both IL-10 and TGF-β haplotypes have indicated remarkable associations with IVDD. While the TGF-β ‘TC’ haplotype (codon 10, codon 25) and IL-10 ‘ACA’ haplotype (− 1082, − 819, − 592) have not been inherited in any of healthy subjects, some of the IVDD patients have inherited these haplotypes. In addition, two other haplotypes of IL-10, ‘ATA’ and ‘ACC’, have been in significant association with disease; while the first one has been 1.69 time more common in patients (*P* = 0.0014), the other one has shown a protective effect as it was more commonly inherited by healthy subjects (*P* < 0.001) (Table 3, Fig. 3).

**Table 3:** IVDD-Control haplotype frequency comparison

Cytokine	Haplotype	IVDD	Control	*P*-Value	OR	95% CI
		(*N* = 76)	(N = 140)			
		N (%)	N (%)			
TGF-β (codon10,codon25)	CG	65 (43)	110 (39.9)	0.54	1.14	0.75–1.71
	TG	73 (48.3)	145 (52.5)	0.42	0.85	0.57–1.26
	CC	9 (6)	21 (7.6)	0.56	0.77	0.34–1.73
	TC	3 (2)	0 (0)	0.04	–	–
IL-10 (−1082,-819,-592)	GCC	58 (38.2)	99 (35.4)	0.60	1.13	0.75–1.69
	ACC	19 (12.5)	100 (35.7)	0.000	0.26	0.15–0.44
	ATA	62 (40.8)	81 (28.9)	0.014	*1.69*	*1.12–2.56*
	ACA	10 (6.6)	0 (0)	0.000	–	–
	GCA	1 (0.7)	0 (0)	0.35	–	–
	ATC	2 (1.3)	0 (0)	0.12	–	–

### Association between age and allele or genotype distributions of IL-10 and TGF-β single nucleotide polymorphisms in IVDD patients

Among all the investigated SNPs, there was a significant association between the average of age and genotype distributions of IL-10 -1082 A/G. Accordingly, the mean age of patients with ‘GG’ genotype was remarkably higher (57.00 ± 2.82) and the mean age of patients with ‘AA’ genotype was lower (37.76 ± 10.14) than the others (*P* = 0.026). This could propose that ‘GG’ genotype could make the individuals more susceptible for IVDD in older ages, while the ‘AA’ genotype could be associated with early onset of disease (Table 4).

**Table 4:** Association of Age and Allele/Genotype Frequencies of IL-10 and TGF-β Single Nucleotide Polymorphisms

Cytokine	Position	Alleles/ Genotypes	IVDD (*N* = 76)	Age ± SD (years)	*P*-Value
			*N* (%)		
TGF-β	Codon 10	C	76 (50)	39.51 ± 10.20	0.61
		T	76 (50)	38.64 ± 11.00	
		CC	13 (17.1)	41.00 ± 8.05	0.77
		CT	50 (65.8)	38.74 ± 11.21	
		TT	13 (17.1)	38.46 ± 11.03	
TGF-β	Codon 25	C	12 (8)	35.08 ± 9.75	0.18
		G	138 (92)	39.30 ± 10.64	
		CC	1 (1.3)	32.00	0.45
		GC	10 (13.3)	35.70 ± 10.66	
		GG	64 (85.3)	39.58 ± 10.67	
IL-10	-1082	A	93 (61.2)	39.40 ± 10.40	0.99
		G	59 (38.8)	39.07 ± 10.95	
		AA	19 (25)	41.00 ± 10.76	**0.026**
		GA	55 (72.4)	37.76 ± 10.14	
		GG	2 (2.6)	57.00 ± 2.82	
IL-10	-819	C	88 (57.9)	39.50 ± 10.45	0.56
		T	64 (42.1)	38.50 ± 10.81	
		CC	20 (26.3)	41.10 ± 9.86	0.58
		CT	48 (63.2)	38.17 ± 10.94	
		TT	8 (10.5)	39.50 ± 11.07	
IL-10	-592	A	73 (48)	38.75 ± 10.82	0.71
		C	79 (52)	39.38 ± 10.42	
		AA	9 (11.8)	38.44 ± 10.93	0.86
		CA	55 (72.4)	38.85 ± 10.98	
		CC	12 (15.8)	40.58 ± 9.31	

### Association between allele/genotype distributions and post-operative visual analogue scale/Oswestry disability index (ODI)

There was a significant association between IL-10 -819C/T genotypes and both 2-month (*P* = 0.04) and 6-month (*P* = 0.03) post-operative VAS; and the ‘CC’ genotype has indicated higher post-operative mean difference among patients. The ‘C’ allele of this SNP has indicated a remarkable 2-month post-operative VAS improvement as well (*P* = 0.05). This may indicate the association between IL-10 ‘C’ allele and response to treatment in IVDD patients. The other investigated SNPs however, failed to show any significant association with post-operative VAS or ODI.

## Discussion

Purposing on clarifying the role of anti-inflammatory cytokines in IVDD, the SNPs of IL-10 and TGF-βincluding IL-10 -1082G/A, IL-10 -819C/T, IL-10 -592A/C, TGF-β Codon 10 C/T, and TGF-β Codon 25 C/T were investigated and compared between IVDD patients and controls through PCR-SSP.

The results of this study indicated significant association between IL-10 allele or genotype distributions and IVDD. The ‘T’ allele of IL-10 -819C/T and the ‘C’ allele of IL-10 -592A/C were more common among patients; and therefore could be considered as the risk alleles. On the other hand, the ‘C’ and ‘A’ alleles of respective SNPs showed protective roles, as their remarkable higher frequency among healthy individuals. Accordingly, the genotypes including ‘CT’ of IL-10 -819C/T, ‘CA’ of IL-10 -592A/C, and ‘GA’ of IL-10 -1082A/G were more common among patients, while the ‘CC’ genotype of both IL-10 -819C/T and IL-10 -592A/CSNPs were more frequent in controls. In addition, the IL-10 haplotypes including ‘ACC’, ‘ATA’, and ‘ACA’ were significantly associated with disease. Interestingly, although none of investigated SNPs of TGF-β was remarkably associated with disease, their co-inheritance (‘TC’ haplotype, codon 10, codon 25) was more common among patients, which might suggest an additive effect of these two polymorphisms in this disease. Besides, the ‘C’ allele and ‘CC’ genotype of IL-10 -819C/T were correlated with response to treatment as indicated better post-operative VAS improvement.

Alterations in IVD matrix components including structural and metabolic components, as well as their interactions, gradually change a healthy disc to a degenerated one. The cytokines were proposed to play important roles in pathophysiology of IVDD, specially the inflammatory ones [9, 24] which are regulated by immunologic regulators as anti-inflammatory cytokines [5]. IL-10 is an anti-inflammatory cytokine with gene location at 1q31-1q32 [5]. The disulfide bonds between two structural cysteines have crucial roles in its proper function, as their inconstancy will lead to structural and subsequently functional dysfunction of this molecule. The molecule has two subunits making its six helix V-shaped form. While IL-10 could have stimulatory effect on NK and B cells, it has inhibitory effects on pro-inflammatory cytokine production and monocyte activity through different pathways [25]. The expression level of IL-10 was remarkably increased in different spine components (bone, disc, ligament) in an animal model of IVDD [7], as similarly detected higher IL-10 expression in Turkish IVDD patients (*P* = 0.0001) [6]. Promoter polymorphisms were found in association with IVDD through alteration in the structure and subsequently function of this immunologic regulator. Compatible with current results, the ‘A’ allele of IL-10 -592A/C was significantly more common among Chinese IVDD patients (*P* = 0.001). Besides, the respective ‘AA’ genotype was also more frequent among patients. As the higher frequency of both ‘G’ allele and ‘GG’ genotype of IL-10 -1082A/G in healthy subjects, this allele could be considered as a protective factor in this population [5]; however, it was not remarkable among Finish population [26]. The IL-10 -819C/T on the other hand, was not associated with disease in Chinese population [5].

Comprising of a large family of anabolic cytokines, TGF-β has different metabolic functions in intervertebral disc matrix including cell regeneration [9], chondrocytes differentiation [10], and matrix calcification [12]. The alteration in TGF-β expression level was detected in both animal models and human subjects with IVDD. Old and completely mature rabbit intervertebral discs indicated higher levels of TGF-β in comparison with the younger or immature ones, more significantly found in annulus fibrosus which was followed by increased levels of other members of this family including BMP-2 and BMP-7 [10, 27]. The results of investigation on human samples were compatible with the animal models, as both mRNA and protein levels of this cytokine were enhanced with degeneration [8, 28]. Moreover, TGF-β expression was in association with level of degeneration and severity of disease as well, when the higher expression level in severe degenerated discs compared with herniated or lower grade ones [11, 13]. An isoform of TGF, specifically found in connective tissue, was more frequently presented in painful discs in comparison with painless or normal tissue [29]. However, a recent investigation reported lower expression levels of TGF-β in higher grades of Thompson classification [12]. On the other hand and compatible with results of current study, the polymorphisms of this anabolic cytokine did not indicate any significant association with disc score or osteophyte formation in Japanese population [30].

Generally, the controversy found in separate genetic investigations in IVDD may support the role of different gene pools in ethnic groups. Besides, the role of other causal factors as mechanical load, age, sex, and other environmental factors should be considered as well in determining the net effect of either IL-10 or TGFβ in pathophysiology of IVDD.

Taking the clinical impact of genetic variants in practical approaches to count, the safety and efficacy of selective cytokine-suppressor medications should be investigated in IVDD patients who would be categorized accordingly to their genotypes. In case of acceptable clinical improvement and cost beneficence of the aforementioned medications, they could be potentially considered as an alternative non-invasive treatment for IVDD instead of surgical intervention.

In the current study, it was attempted to consider a precise inclusion criteria and exclude all those who were affected with other inflammatory or immune-related diseases which could confound the results of the study. Moreover, a link was created between genetic variants in IVDD patients and demographic (exe. age) or clinical (pain reduction after surgery) characteristics. This could be considered a forward step to create links between basic science and clinic. Meanwhile, the statistical power of the study was 80% which is acceptable in many statistical analysis including those applied in the current study. However, much larger sample size is required for further epidemiologic investigations. On the other hand, the patients included all adult patients ranging from 19 to 62 years, which could probably affect the results as the disc degeneration is an aging process. Moreover, the other important factors in genetic investigations should be considered in the future studies. Therefore, a prospective cohort study with a robust methodology and high statistical power should be designed to include homogenous and matched population in order to evaluate the suspicious genetic risk factors. It is highly recommended to have careful considerations regarding potential confounders such as physical activity, job, etc. A multivariate analysis would be useful as well in order to assess additive effect of causal factors in IVDD. Meanwhile, as the inherited SNP variants were of interest in this study, the age of subjects was not considered as a causative factor for SNP variant. However, as the possible role of somatic mutations in pathology of IVDD, it is recommended to consider the SNP investigation in disc tissues in age-matched groups.

## Conclusion

The inflammatory cytokines, as well as their modulators alter in the process of disc degeneration; and therefore, their polymorphisms could have causal effects on pathophysiology of this disease. Promoter polymorphisms of IL-10 have been significantly found in association with IVDD in the current study; however, the role of TGF-β was not remarkable in Iranian IVDD patients.

## Abbreviations

IL-10: Interleukin 10
IVDD: Intervertebral Disc Degeneration
MD: Mean Differences
ODI: Oswestry disability index
OR: Odds Ratio
PCR: Polymerase Chain Reaction
PCR-SSP: Sequence Specific Prime PCR
SNP: Single Nucleotide Polymorphisms
TGF-β: Transforming Growth Factor β
VAS: Visual Analogue Scale
